# Single-port compared with conventional laparoscopic ovarian cystectomy for benign ovarian cysts: a systematic review and meta-analysis

**DOI:** 10.3389/fonc.2026.1704981

**Published:** 2026-06-09

**Authors:** Yuhao Zhu, Rong Tang, Lingli Hu, Yupei Lei, Weimin Xie

**Affiliations:** 1 Department of Acupuncture and Moxibustion, Heilongjiang University of Chinese Medicine, Harbin, Heilongjiang, China; 2 Department of Gynecology, Affiliated Nanhua Hospital, Hengyang Medical School, University of South China, Hengyang, Hunan, China; 3 Department of Ultrasound, Affiliated Hengyang Hospital of Hunan Normal University & Hengyang Central Hospital, Hengyang, Hunan, China; 4 Department of Obstetrics, Affiliated Hengyang Hospital of Hunan Normal University & Hengyang Central Hospital, Hengyang, Hunan, China; 5 Department of Gynecology, Affiliated Hengyang Hospital of Hunan Normal University & Hengyang Central Hospital, Hengyang, Hunan, China

**Keywords:** cystectomy, laparoscopy, ovarian cysts, single-port, systematic review

## Abstract

**Objective:**

To evaluate current evidence on the safety and impact on ovarian reserve of single-port laparoscopic cystectomy (SLC) for the treatment of benign ovarian cysts.

**Methods:**

We comprehensively searched PubMed, Embase, the Cochrane Library, and ClinicalTrials.gov from their inception to 31 December 2025. We included randomized controlled trials (RCTs) or non-randomized studies (NRSs) comparing SLC with conventional laparoscopic cystectomy (CLC) for benign ovarian cysts. The primary outcomes were postoperative changes in serum anti-Müllerian hormone (AMH) concentration, intraoperative complications, postoperative complications, and cosmetic outcomes. All analyses were performed using random-effects or fixed-effects models.

**Results:**

In total, 14 full-text studies involving 1, 691 participants were included in the final analysis. There were no significant differences between SLC and CLC in the incidence of intraoperative complications (*P* = 0.50), postoperative complications (*P* = 0.49), postoperative changes in serum AMH concentration (*P* = 0.06), estimated blood loss (*P* = 0.99), hemoglobin loss (*P* = 0.76), time to flatus (*P* = 0.68), length of hospital stay (*P* = 0.17), or analgesic use (*P* = 0.91). SLC was associated with significantly lower postoperative pain scores at 24 hours (*P* = 0.02) but longer operative time (*P* = 0.001) compared with CLC.

**Conclusion:**

This systematic review and meta-analysis provides evidence that SLC may be offered as a minimally invasive surgical alternative for women with benign ovarian cysts, at the cost of a longer operative time compared with CLC. Given the retrospective design of the majority of the included studies, further large-scale randomized controlled trials are warranted to validate these findings.

**Systematic review registration:**

https://www.crd.york.ac.uk/PROSPERO/view/, identifier CRD42024602477.

## Introduction

Ovarian cysts are common gynecological disorders, the majority of which are benign, and are most prevalent among women of childbearing age. The mainstay of treatment for symptomatic benign ovarian cysts is surgical management. For premenopausal patients, ovarian cystectomy is widely performed to preserve ovarian function. Laparoscopic surgery has become a popular alternative to laparotomy due to its many advantages, including shorter hospital stay, faster recovery, and less postoperative pain (1). Consequently, laparoscopic ovarian cystectomy is currently considered the gold standard surgical management for women with benign ovarian cysts (2).

Recently, transumbilical single-port laparoscopy, also known as laparoendoscopic single-site surgery, has been developed in gynecological surgery to enhance cosmetic benefits (3). In the surgical treatment of benign ovarian cysts, single-port laparoscopic cystectomy (SLC) has also been reported as an alternative to conventional laparoscopic cystectomy (CLC); however, the results remain controversial (4–6). Since most women requiring ovarian cystectomy are relatively young and wish to preserve their fertility potential, it is critical to evaluate the impact of laparoscopic ovarian cystectomy itself on ovarian reserve. Given that single-port laparoscopy has certain limitations, such as restricted instrument mobility, SLC may be more difficult to perform than CLC and could have a greater negative impact on ovarian reserve. Therefore, the role of SLC in women with benign ovarian cysts remains to be established.

Although several markers of ovarian reserve exist, circulating anti-Müllerian hormone (AMH) is now widely accepted as one of the most reliable (7). Serum AMH concentration is generally stable throughout the menstrual cycle with minimal variations, making it an ideal marker for detecting relatively small changes in ovarian reserve following ovarian cystectomy (8). Therefore, this systematic review and meta-analysis assessed the impact on ovarian reserve using anti-Müllerian hormone (AMH).

Based on the above, we designed this systematic review and meta-analysis to systematically search for and analyze available studies comparing the safety and impact on ovarian reserve of SLC versus CLC for benign ovarian cysts. Although this systematic review and meta-analysis focuses on benign ovarian cysts, it is crucial to acknowledge their oncological implications during surgical management. Specifically, intact resection of ovarian cysts and avoidance of intraoperative spillage are of great importance, as these measures can effectively prevent tumor upstaging in cases where unexpected borderline or malignant neoplasms are identified intraoperatively.

## Materials and methods

The protocol for this systematic review and meta-analysis was registered in the International Prospective Register of Systematic Reviews (PROSPERO) on 25 January 2025 (registration number: CRD42024602477). This systematic review was conducted in accordance with the Preferred Reporting Items for Systematic Reviews and Meta-analyses (PRISMA) Statement (9) and the Assessing the Methodological Quality of Systematic Reviews (AMSTAR) standards (10).

### Literature search

PubMed, Embase, the Cochrane Library, and ClinicalTrials.gov were searched from their inception to 31 December 2025. The following search strategy was used: (single incision OR single access OR single site OR single port OR single trocar) AND (laparoscop* OR laparoendoscop* OR coelioscop* OR celioscop* OR peritoneoscop* OR abdominoscop*) AND (ovarian cystectomy). To broaden the search, we also screened the reference lists of the included articles for additional studies. No language restrictions were applied.

### Selection criteria and eligibility criteria

Two independent reviewers removed duplicate records and independently screened titles and abstracts to identify potentially relevant studies. Full-text articles of potentially relevant studies were screened against the inclusion and exclusion criteria. Any disagreements during the literature screening process were resolved through discussion under the supervision of a third reviewer.

The inclusion criteria were as follows: (1) population: women with benign ovarian cysts; (2) intervention: transumbilical single-port laparoscopic ovarian cystectomy; (3) comparator: conventional laparoscopic ovarian cystectomy; (4) outcomes: perioperative outcomes; (5) study design: randomized controlled trials (RCTs) or non-randomized studies (NRSs).

The exclusion criteria were as follows: (1) abstracts, letters, editorials, expert opinions, case reports, comments, reviews, and book chapters; (2) studies without comparisons; (3) studies assessing perioperative outcomes of surgeries other than those related to ovarian cystectomy; (4) studies without appropriate data for the selected outcomes; (5) duplicate studies.

### Outcomes of interest

The following outcomes were used to compare SLC with CLC. The primary outcomes were postoperative changes in serum AMH concentration, intraoperative complications, postoperative complications, and cosmetic outcomes. The secondary outcomes were operative time (min), estimated blood loss (mL), hemoglobin loss (g/L), time to flatus (d), length of hospital stay (d), conversion to laparotomy, postoperative pain scores, and analgesic use.

### Quality assessment

The methodological quality of the non−randomized studies was independently assessed by two authors using the Newcastle–Ottawa Scale (NOS) (11). The NOS evaluates study quality using nine items across three categories: selection, comparability, and outcomes (for cohort studies) or exposure (for case−control studies). One point was assigned to each item judged as low risk, and a total score was calculated, ranging from 0 (lowest quality) to 9 (highest quality). The tool recommended by the Cochrane Collaboration was used to evaluate the methodological quality of the RCTs included in this meta−analysis (12). The risks of selection bias, performance bias, detection bias, attrition bias, reporting bias, and other bias were classified as “low”, “high”, or “unclear”. If the two authors could not reach a consensus, a third author was consulted to arbitrate and provide a definitive inclusion decision.

### Data extraction and analysis

Two authors manually extracted the following data from the eligible studies into spreadsheets: general study information, participant age, body mass index (BMI), study population size, interventions, comparators, and outcomes. When data were unavailable in a publication, the study authors were contacted to obtain the missing details.

Risk ratios (RRs) with 95% confidence intervals (CIs) were used to measure dichotomous outcomes, whereas weighted mean differences (WMDs) with 95% CIs were used to compare continuous outcomes. To calculate the RR, the total number of patients in each group and the number of patients with the event of interest were extracted from each study; for continuous outcomes, the mean and standard deviation (SD) were extracted to calculate the effect measure. If continuous data were expressed as median and range, we calculated the mean and SD. Statistical heterogeneity across studies was assessed using the chi−squared test and the I² statistic. Heterogeneity was considered high if *P* < 0.10 or I² > 50%, and low if *P* ≥ 0.10 or I² ≤ 50%. Raw data from the selected studies were pooled using a fixed−effects model when heterogeneity was low, whereas a random−effects model was applied when heterogeneity was high. Sensitivity analyses were performed by omitting each study iteratively to assess the robustness of the pooled results. If more than 10 eligible studies were included, potential publication bias was assessed using funnel plots according to the Cochrane Handbook of Systematic Reviews. All analyses were conducted using RevMan version 5.3.5 and Stata version 17.0.

## Results

**Figure 1:**
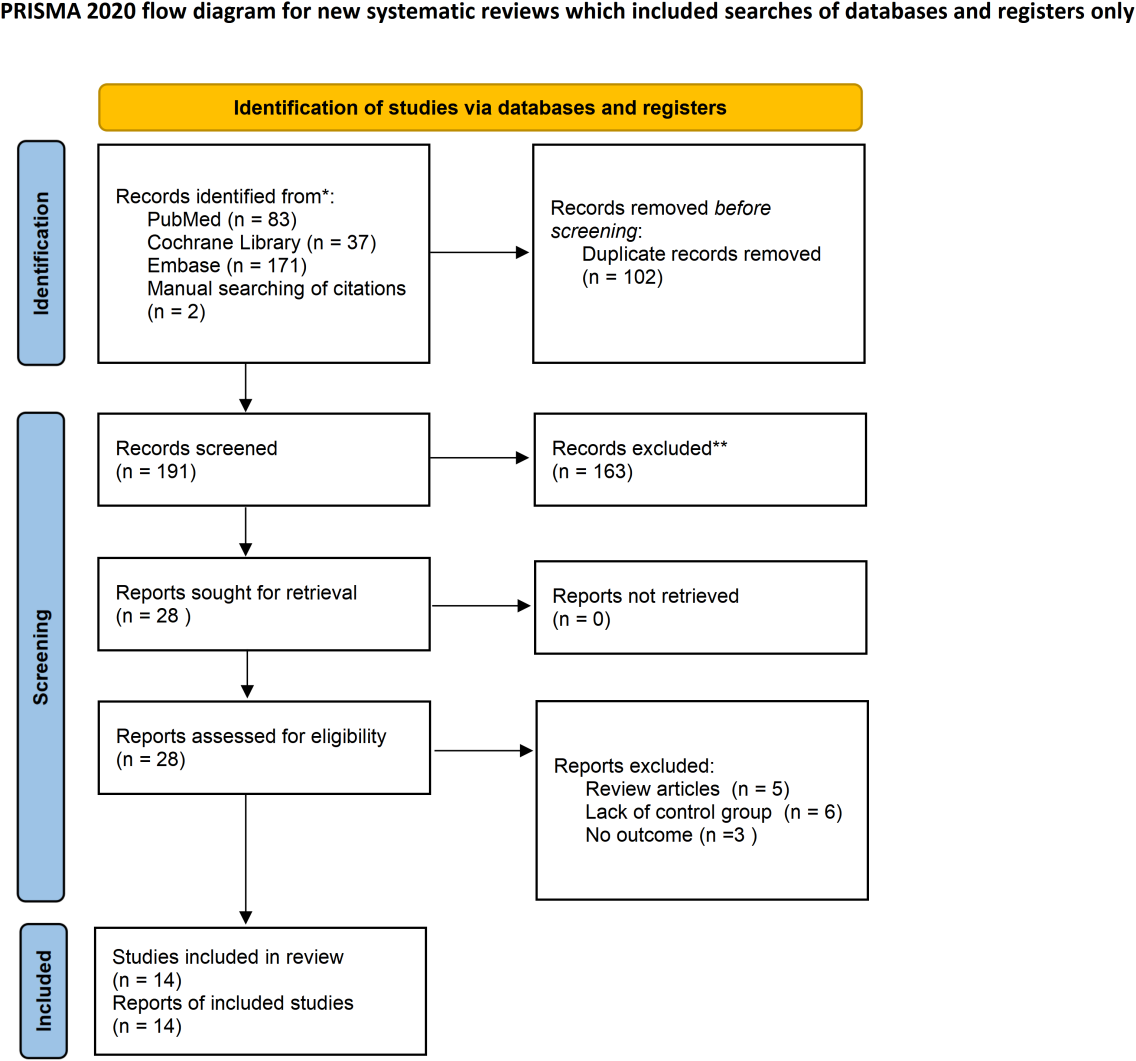
PRISMA flow diagram of the study selection.

From the initial search, 291 records were obtained from PubMed (n = 83), the Cochrane Library (n = 37), and Embase (n = 171). The manual searching of citations added another two records. After removing duplicate records, 191 records were screened by title, resulting in 119 articles for abstract screening. Following the exclusion of 91 records, the remaining 28 articles were selected for full-text review. Among these, a comprehensive assessment excluded 14 records for various reasons, as illustrated in **Figure 1**. Consequently, 14 full-text studies matched the inclusion criteria and were included in this systematic review and meta-analysis (4–6 and 13-23). The PRISMA flow diagram for the systematic search and selection of articles for this review and meta-analysis is shown in **Figure 1**.

The basic characteristics of the included studies are presented in **Table 1**. A total of 13 articles were NRSs (4–6, 13–21, 23), and the remaining one was an RCT (22). Study sizes ranged from 54 to 343 total patients. The total eligible population was 1, 691, with 863 in the SLC group and 828 in the CLC group. Six studies were conducted in China (6, 15, 17, and 19-21), four in South Korea (13, 16, 18, 22), one in Italy (4), one in the United States (5), one in Taiwan (14), and one in Türkiye (23). With regard to the quality assessment, the NOS scores of the 13 included NRS studies ranged from 7 to 9 points. For the only RCT comparing SLC with CLC, the risk of selection bias, attrition bias, and reporting bias was low, whereas the risk of performance bias, detection bias, and other bias was unclear. Randomization was achieved through the use of sealed opaque envelopes in random blocks previously prepared by a statistician. The envelope containing the group allocation was opened in the operating room by a study nurse, who was not involved in the randomization procedure, before surgery, allowing the operating equipment to be prepared. This study was open-label.

**Table 1:** The basic characteristics of the included studies.

Author (year)	Location	Study type	Sample size	Cyst size, cm	Age (year)	BMI (kg/m^2^)	Previous abdominal surgery	Study quality (NOS scores)
Angioni et al. (2015) (4)	Italy	Prospective case-control study	SLC:49 CLC:50	7.6 ± 3.56 7.0 ± 2.0	30.25 ± 5.1 30.53 ± 4.55	20.42 ± 2.3 20.19 ± 2.58	NA	8
Bedaiwy et al. (2015) (5)	United States	Retrospective cohort study	SLC:31 CLC:57	6.5 ± 2.1 7.2 ± 2.1	32 ± 5 34 ± 5	27 ± 3 28 ± 4	11 (35.4) 19 (33.3)	9
Chen et al. (2022) (6)	China	Retrospective cohort study	SLC:123 CLC:187	NA	30.36 ± 7.36 30.88 ± 6.25	21.44 ± 3.77 21.58 ± 3.22	NA	8
Chong et al. (2015) (13)	South Korea	Retrospective case-control study	SLC:25 CLC:33	11.4 ± 4.2 9.7 ± 2.3	23.3 ± 8.3 22.3 ± 4.5	21.1± 3.1 22.5 ± 3	NA	7
Huang et al. (2014) (14)	Taiwan	Retrospective case-control study	SLC:34 CLC:37	7.05 ± 2.37 6.39 ± 2.27	34.59 ± 10.18 34.73 ± 9.68	21.29 ± 2.52 21.14 ± 2.61	7 (20.6) 4 (10.8)	9
Jiang et al. (2023) (15)	China	Retrospective cohort study	SLC:37 CLC:45	5.86 ± 3.24 5.91 ± 1.94	31.05 ± 8.28 34.11 ± 7.32	21.93 ± 3.37 22.46 ± 3.10	14 (37.8) 15 (33.3)	9
Kim et al. (2019) (16)	South Korea	Retrospective cohort study	SLC:216 CLC:38	7.22 (1.3-67.7) 7.51 (3.4-17.8)	29.2 (8-49) 31.6 (10-47)	21.5 (14.3-38.4) 22.7 (17.2-31.5)	21 (9.7) 6 (15.8)	7
Liu et al. (2017) (17)	China	Retrospective cohort study	SLC:40 CLC:41	6.3 ± 2.6 6.5 ± 2.3	30.4 ± 7.0 29.3 ± 5.0	21.8 ± 3.5 21.9 ± 3.8	9 (23) 9 (22)	9
Park et al. (2015) (18)	South Korea	Retrospective cohort study	SLC:154 CLC:189	5.7 ± 2.1 6.1 ± 2.1	35.3 ± 6.9 33.4 ± 7.1	21.74 ± 3.07 21.25 ± 2.88	28 (18.2) 40 (21.2)	8
Wang et al. (2019) (19)	China	Non-randomized concurrent control trial	SLC:47 CLC:32	6.4 ± 2.3 5.9 ± 1.6	30.1 ± 6.8 29.4 ± 5.1	20.8 ± 3.3 20.6 ± 2.7	NA	9
Wang et al. (2021) (20)	China	Retrospective cohort study	SLC:33 CLC:27	17.36 ± 4.07 16.33 ± 2.09	31.58 ± 11.73 32.44 ± 12.53	25.09 ± 4.56 23.6 ± 4.25	0.15 ± 0.36 0.29 ± 0.47	8
Xiong et al. (2014) (21)	China	Prospective cohort study	SLC:24 CLC:30	NA	31 ± 9 31 ± 10	19.1 ± 2.1 19.7 ± 2.3	NA	7
Yoon et al. (2014) (22)	South Korea	Randomized controlled trial	SLC:28 CLC:29	6.1 ± 1.9 6.5 ± 2.3	30.3 ± 5.0 28.8 ± 5.2	20.4 ± 2.4 20.4 ± 2.4	4 (13.8) 7 (24.1)	–
Ülker et al. (2013) (23)	Türkiye	Prospective cohort study	SLC:22 CLC:33	8.207 ± 4.364 7.548 ± 2.803	37.22 ± 13.35 38.67 ± 12.47	24.97 ± 4.72 25.41 ± 4.89	10 (45.4) 14 (42.4)	8

Values are presented as means ± SD or n (%). SLC, single-port laparoscopic cystectomy; CLC, conventional laparoscopic cystectomy; BMI, body mass index; NOS, Newcastle-Ottawa Scale; NA, not available.

### Primary outcomes

**Figure 2:**
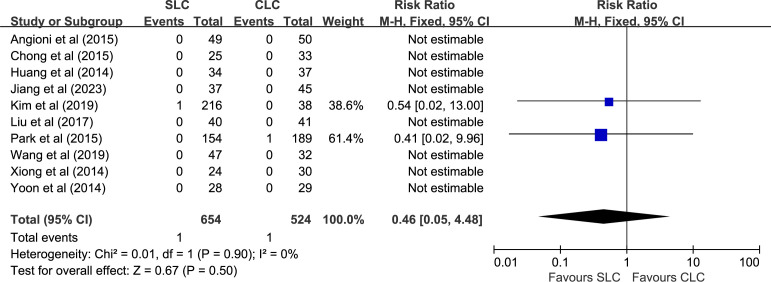
Forest plot comparing intraoperative complications between SLC and CLC.

A total of 10 studies (4, 13–19, 21, 22) reported intraoperative complications. Pooled data using the fixed-effects model showed that the incidence of intraoperative complications was similar between the SLC and CLC groups (RR, 0.46; 95% CI, 0.05 to 4.48; *P* = 0.50) (**Figure 2**). The data were homogeneous across studies (*P* = 0.90, I^2^ = 0%). The only RCT (22) reported no cases of intraoperative complications in either group.

**Figure 3:**
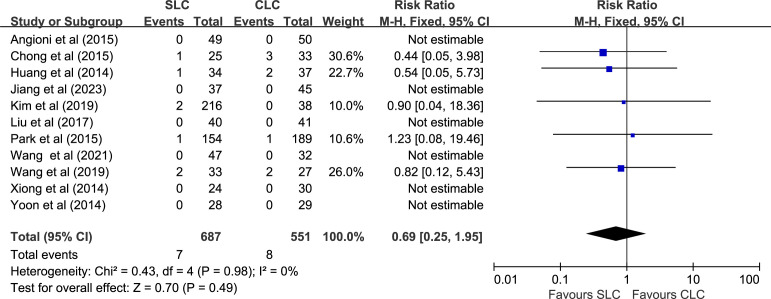
Forest plot comparing postoperative complications between SLC and CLC.

Postoperative complications were investigated in 11 studies (4, 13–22). The incidence of postoperative complications was similar between the two groups (RR, 0.69; 95% CI, 0.25 to 1.95; *P* = 0.49). The pooled analysis showed no heterogeneity (*P* = 0.98, I^2^ = 0%) (**Figure 3**). The only RCT (22) reported no cases of postoperative complications in either group.

**Figure 4:**
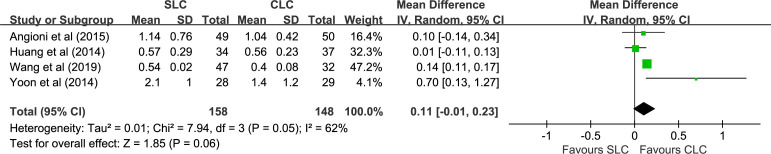
Forest plot comparing postoperative changes in serum AMH concentrations between SLC and CLC.

Four studies (4, 14, 19, 22) investigated the impact on ovarian reserve using AMH. In all four included studies, a decline in serum AMH concentration was observed at 1 month after laparoscopic ovarian cystectomy for benign ovarian cysts. Pooled analysis of the included studies revealed no statistically significant change in serum AMH concentrations between the two groups after ovarian cystectomy (WMD, 0.11; 95% CI, -0.01 to 0.23; *P* = 0.06), with high heterogeneity across studies (*P* = 0.05, I^2^ = 62%) (**Figure 4**). The only RCT (22) reported no significant difference in serum AMH concentrations at postoperative sampling points.

Three studies (15, 17, 21) reported on cosmetic outcomes, but this outcome was not analyzed in the meta-analysis because the reporting was not standardized across the included studies. Jiang et al. (15) measured satisfaction scores using Kiyak’s satisfaction rating scale and showed that the satisfaction score was significantly higher in the SLC group than in the CLC group. Liu et al. (17) used the validated Body Image Questionnaire and showed that patients in the SLC group were significantly more satisfied with their scars and had higher body image satisfaction compared with those in the CLC group. Xiong et al. (21) assessed cosmetic outcome satisfaction two months after surgery and demonstrated that patient−evaluated cosmetic satisfaction scores were significantly higher in the SLC group than in the CLC group.

### Secondary outcomes

**Figure 5:**
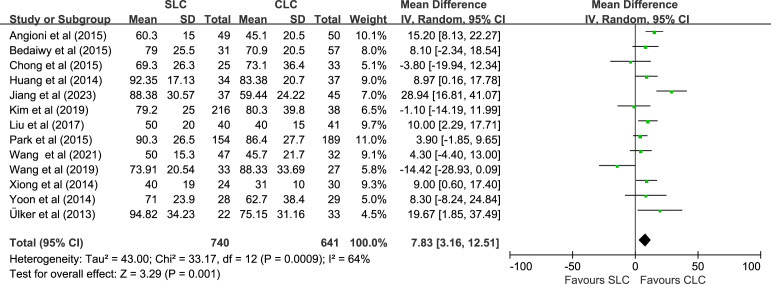
Forest plot comparing operative time between SLC and CLC.

A total of 13 studies (4, 5, 13–23) reported operating time. The combined WMD showed a significantly longer operating time in patients who underwent SLC than in those who underwent CLC (WMD, 7.83; 95% CI, 3.16 to 12.51; *P* = 0.001). Heterogeneity was present across the studies (*P* = 0.0009; I^2^ = 64%) (**Figure 5**). The only RCT (22) reported that operating time was similar between the two groups.

**Figure 6:**
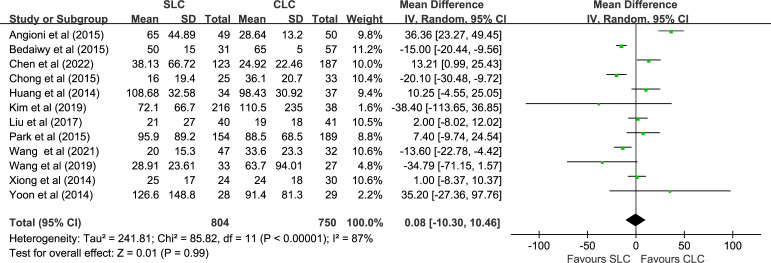
Forest plot comparing estimated blood loss between SLC and CLC.

In all, 12studies (4–6, 13, 14, 16–22) reported estimated blood loss. The estimated blood loss was similar between the two groups (WMD, 0.08; 95% CI, -10.30 to 10.46; *P* = 0.99). The data were heterogeneous across the studies (*P* < 0.00001; I^2^ = 87%) (**Figure 6**). The only RCT (22) reported no significant difference in estimated blood loss between the two groups.

**Figure 7:**
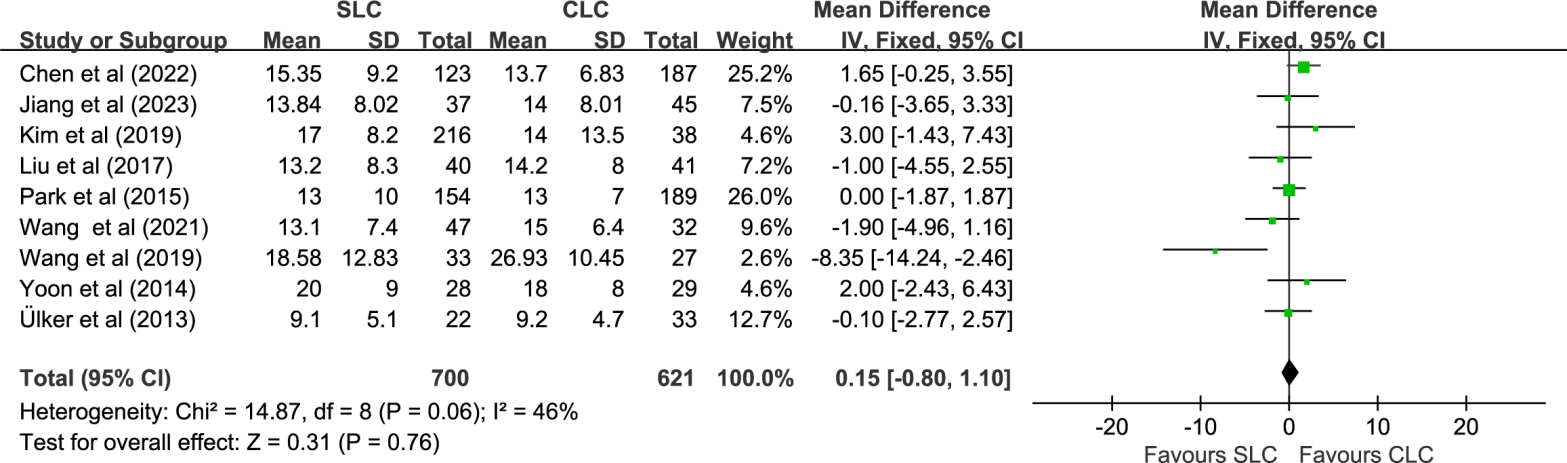
Forest plot comparing hemoglobin loss between SLC and CLC.

Nine studies (6, 15–20, 22, 23) reported data on hemoglobin loss, and the pooled results showed no difference between the two groups (WMD, 0.15; 95% CI, -0.80 to 1.10; *P* = 0.76). No significant heterogeneity was detected across the studies (*P* = 0.06, I^2^ = 46%) (**Figure 7**). The only RCT (22) reported no significant difference in hemoglobin loss between the two groups.

**Figure 8:**
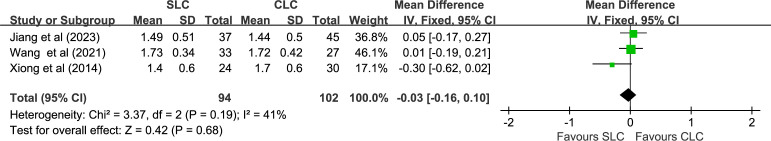
Forest plot comparing time to flatus between SLC and CLC.

Three studies (15, 20, 21) reported data on time to flatus. No difference in time to flatus was observed between the two groups (WMD, -0.03; 95% CI, -0.16 to 0.10; *P* = 0.68). Heterogeneity across the studies was not significant (*P* = 0.19, I^2^ = 41%) (**Figure 8**).

**Figure 9:**
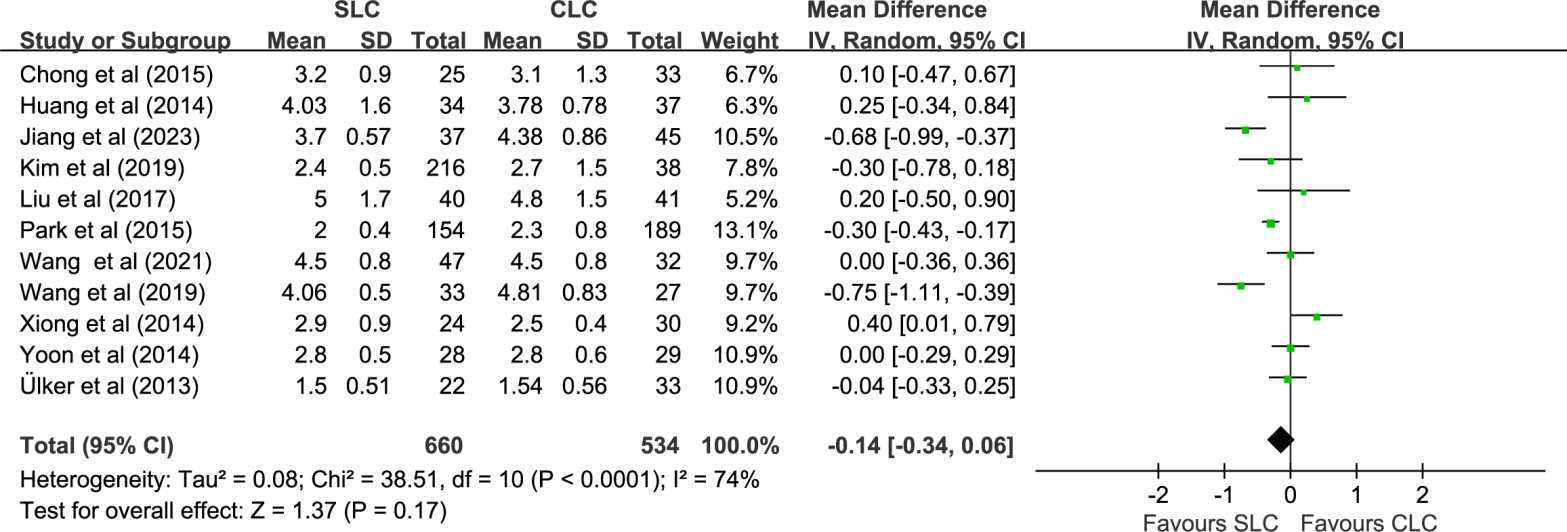
Forest plot comparing length of hospital stay between SLC and CLC.

Eleven studies (13–23) reported data on length of hospital stay. Significant heterogeneity was observed across the studies (*P* < 0.0001, I^2^ = 74%). Pooled analysis using a random-effects model showed no significant difference in length of hospital stay between the two groups (WMD, -0.14; 95% CI, -0.34 to 0.06; *P* = 0.17) (**Figure 9**). The only RCT (22) reported that there was no significant difference in the length of hospital stay between the two groups.

Eight studies (13, 15–20, 22) reported data on conversion to laparotomy. However, a meta−analysis could not be performed because no patient in either group required conversion to laparotomy.

**Figure 10:**
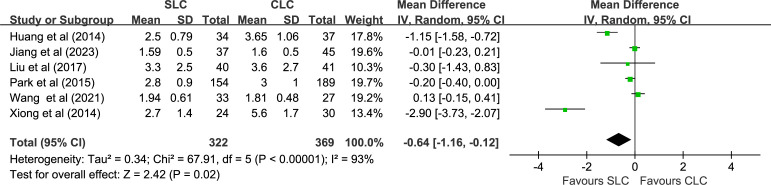
Forest plot comparing postoperative pain scores at 24 h between SLC and CLC.

Six studies (14, 15, 17, 18, 20, 21) reported postoperative pain scores at 24 h. SLC was associated with significantly lower pain scores at 24 h postoperatively compared with CLC (WMD, -0.64; 95% CI, -1.16 to -0.12; *P* = 0.02). Substantial heterogeneity was observed among the studies (*P* < 0.00001; I^2^ = 93%) (**Figure 10**).

**Figure 11:**
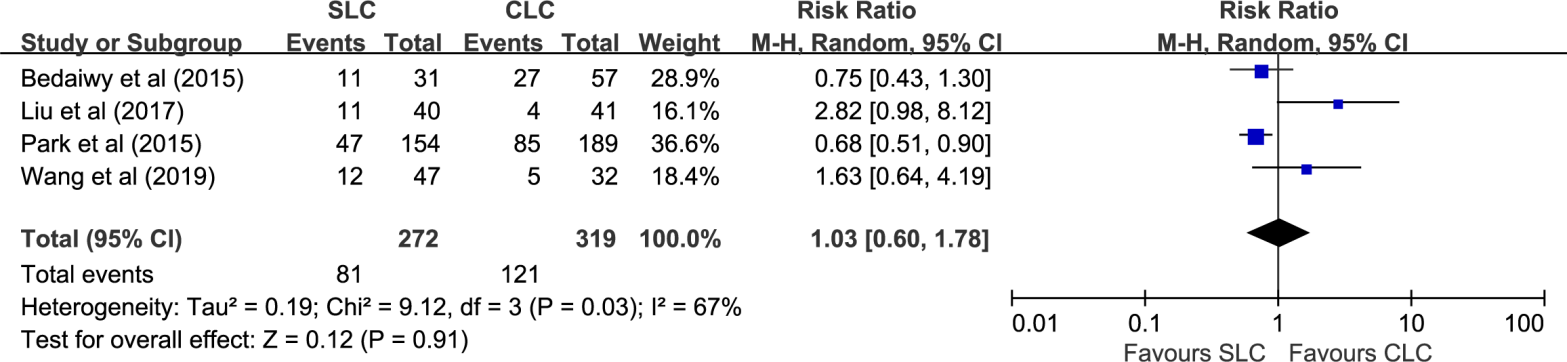
Forest plot comparing analgesic use between SLC and CLC.

Four studies (5, 17–19) reported data on analgesic use. Pooled analysis showed no significant difference in analgesic use between the two groups (RR, 1.03; 95% CI, 0.60 to 1.78; *P* = 0.91). Significant heterogeneity was observed across the studies (*P* = 0.03; I^2^ = 67%) (**Figure 11**).

### Sensitivity analysis

In the sensitivity analysis, each study was sequentially excluded, and the influence of its removal was assessed by recalculating the pooled RR or WMD for the remaining studies. The analysis confirmed the robustness of the results, as no single study substantially altered the pooled effect.

### Publication bias

**Figure 12:**
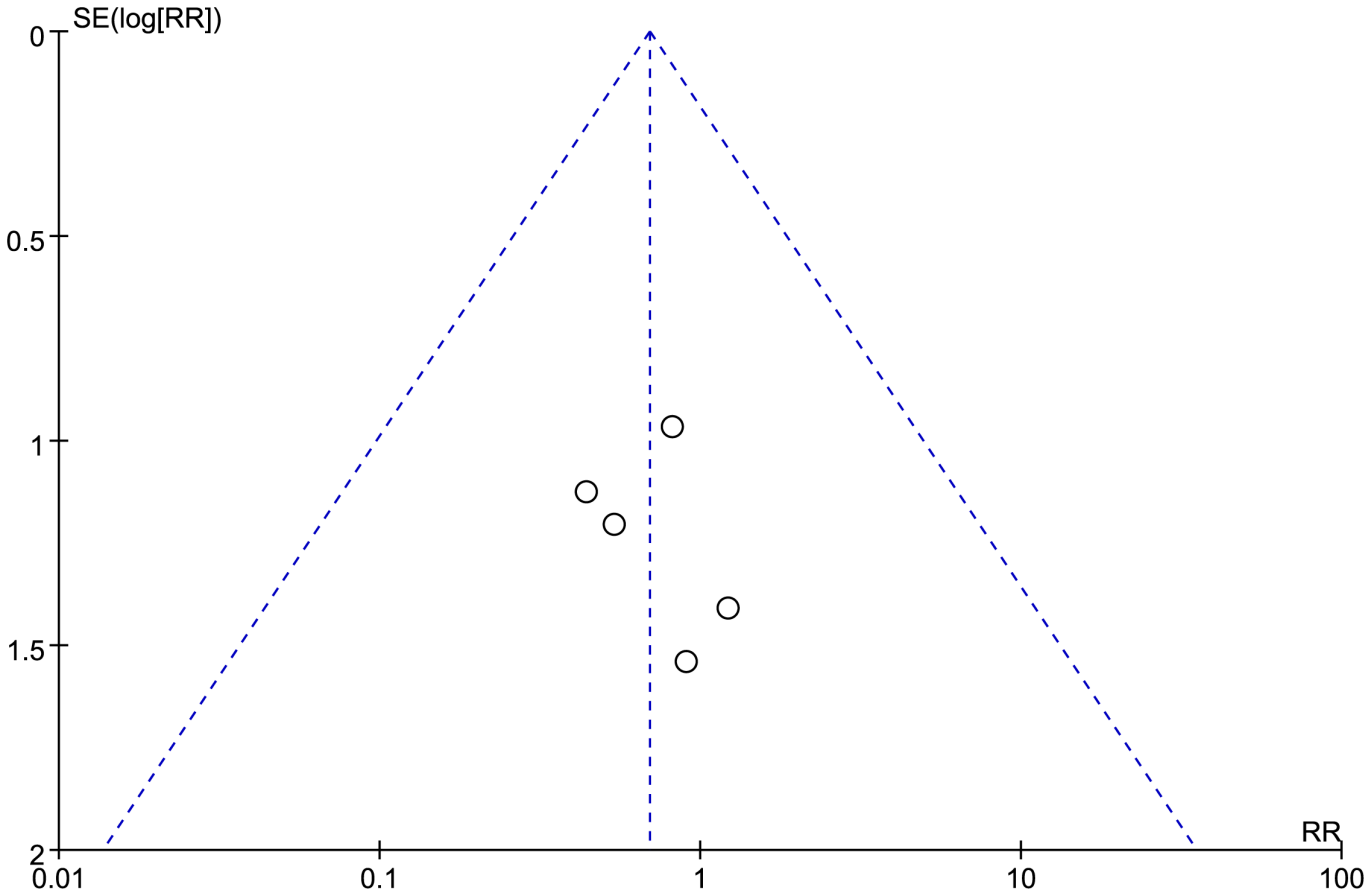
Funnel plot for postoperative complications.

The funnel plot for postoperative complications did not show obvious asymmetry (**Figure 12**). All studies fell within the funnel and were symmetrically distributed, indicating no apparent publication bias in our meta-analysis. No evidence of publication bias for postoperative complications was detected by either the Begg’s or Egger’s test (Begg’s test: *P* = 0.81; Egger’s test: *P* = 0.93).

## Discussion

This systematic review and meta-analysis included 13 NRSs and one RCT comparing SLC with CLC in terms of perioperative outcomes and the impact on ovarian reserve following ovarian cystectomy. Our findings confirmed that SLC was associated with lower postoperative pain scores at 24 h but was generally equivalent to CLC with respect to the incidence of intraoperative complications, postoperative complications, postoperative changes in serum AMH concentration, estimated blood loss, hemoglobin loss, exhaust time, length of hospital stay, and analgesic use. Moreover, operative time was significantly longer in the SLC group.

Safety and efficacy are two of the most important considerations when developing a new surgical technique. The demand for fewer incisions and scarless procedures has driven the application of single-port laparoscopic ovarian cystectomy. In this systematic review and meta-analysis, we demonstrate that SLC is as safe as CLC for treating patients with benign ovarian cysts. Among the included studies, the incidence of intraoperative complications was low in both the SLC and CLC groups, and the pooled data showed no significant difference. The rate of postoperative complications also did not differ between the two groups, and the results showed homogeneity across studies. These results are consistent with a previous meta-analysis comparing single-port laparoscopic hysterectomy with conventional laparoscopic hysterectomy for benign gynecologic conditions (24).

A decrease in AMH may indicate diminished ovarian reserve and, consequently, an earlier onset of menopause (25). In all four included studies, a decline in serum AMH concentration was observed after laparoscopic ovarian cystectomy. The reasons for this negative impact on ovarian reserve have been attributed to the removal of healthy ovarian cortex surrounding the cyst or ovarian damage during hemostasis (26). Compared with conventional laparoscopic surgery, surgeons often find single-port laparoscopy more challenging due to reduced visualization, loss of triangulation, and instrument interference (19, 27). It has been presumed that these limitations make meticulous dissection more difficult than in conventional laparoscopic surgery, potentially leading to a greater negative impact on ovarian reserve during ovarian cystectomy. In this meta-analysis, our findings revealed no significant differences in the change in serum AMH concentrations after ovarian cystectomy between the SLC and CLC groups.

Cosmetic outcome is an important consideration, especially in young female patients with benign ovarian cysts. Nonetheless, only three of the included studies evaluated postoperative wound healing, and all three showed that patients were more satisfied with the cosmetic outcome after single-port laparoscopic ovarian cystectomy than after the multiport approach. Further studies are needed to clarify whether single-port laparoscopy improves cosmetic outcomes or whether a larger umbilical incision is associated with higher incidences of keloids and hernias.

The meta-analysis revealed no significant differences between the two groups in terms of estimated blood loss, hemoglobin loss, time to flatus, length of hospital stay, and analgesic use, demonstrating that single-port laparoscopic ovarian cystectomy is a feasible procedure for benign ovarian cysts. Our data confirm that single-port laparoscopic ovarian cystectomy requires a longer operative time, and the difference between the surgical techniques is both statistically and clinically significant, with a median difference of approximately 7.83 minutes. This indicates increased operative difficulty in single-port laparoscopy due to reduced visualization, loss of triangulation, and instrument interference (19). Furthermore, single-port laparoscopic ovarian cystectomy involves several complex maneuvers, such as complete mass removal, suturing, and knot tying (15). In contrast, a significant reduction in postoperative pain scores at 24 h was reported for patients undergoing single-port laparoscopy, although this benefit does not appear clinically relevant for this endpoint.

This systematic review and meta-analysis has certain limitations. First, most of the included studies were NRSs by design, which may introduce selection bias and thus affect the reliability of the outcome estimates. The pooled WMD showed a significantly longer operative time in patients who underwent SLC than in those who underwent CLC. However, the only RCT (22) reported that operative time was similar between the two groups. Therefore, this finding should be interpreted with caution. Second, significant heterogeneity was observed for some outcomes, which lowers the confidence in the estimates. To explore sources of heterogeneity, we conducted a comprehensive sensitivity analysis. This analysis confirmed that the pooled effect sizes for the outcomes remained stable, indicating that the random-effects estimates were robust and not unduly influenced by individual studies. This significant heterogeneity likely reflects clinical diversity, including differences in surgical protocols, baseline patient characteristics, cyst pathology, anesthesia regimens, and methods for measuring estimated blood loss. Third, the included studies did not provide appropriate data to evaluate cosmetic outcomes. Fourth, a notable limitation of this meta-analysis is the lack of available data in the included studies to distinguish between ovarian endometriomas and non-ovarian endometriomas cysts. Different types of benign ovarian cysts have significantly distinct impacts on ovarian reserve, with endometriomas often exerting more pronounced effects on indicators such as AMH compared with other cyst types. Finally, the follow-up periods of the included studies were generally short, so long-term clinical outcomes—such as cosmetic results, port-site hernia, and the long-term impact on ovarian reserve—remain to be further explored. Therefore, future research should aim to conduct more large-scale prospective randomized trials with long-term follow-up to substantiate and reinforce the conclusions drawn in this study.

## Conclusions

This systematic review and meta-analysis supports the use of SLC in women with benign ovarian cysts, as it is associated with lower postoperative pain scores at 24 hours and comparable outcomes to CLC in terms of intraoperative and postoperative complications, postoperative changes in serum AMH concentration, estimated blood loss, hemoglobin loss, time to flatus, length of hospital stay, and analgesic use. Notably, operative time appears longer with SLC than with CLC. SLC is a safe and feasible technique that may be offered as an alternative to CLC for selected patients, given its potential benefits, including better cosmetic outcomes. Further studies, including multicenter prospective randomized controlled trials with long-term follow-up, are required to better assess all aspects of SLC.

## Data Availability

The original contributions presented in the study are included in the article/**Supplementary Material**. Further inquiries can be directed to the corresponding author/s.

## Supplementary material

Supplementary Material 1 PRISMA 2020 checklist.
